# Supplementary material for: Corticohippocampal circuit dysfunction in a mouse model of Dravet syndrome
Source: eLife. 2022 Feb 25;11:e69293. doi: 10.7554/eLife.69293 (PMC8920506; doi:10.7554/eLife.69293)
Supplement: Transparent reporting form [file elife-69293-transrepform1.pdf]

eLIFE
1st Floor
24 Hills Road
Cambridge CB2 1JP, UK
P 01223 855340
W elifesciences.org
T @elife

eLife's transparent reporting form

We encourage authors to provide detailed information *within their submission* to facilitate the interpretation and replication of experiments. Authors can upload supporting documentation to indicate the use of appropriate reporting guidelines for health-related research (see EQUATOR Network), life science research (see the BioSharing Information Resource), or the ARRIVE guidelines for reporting work involving animal research. Where applicable, authors should refer to any relevant reporting standards documents in this form.

If you have any questions, please consult our Journal Policies and/or contact us: editorial@elifesciences.org.

Sample-size estimation

- You should state whether an appropriate sample size was computed when the study was being designed
- You should state the statistical method of sample size computation and any required assumptions
- If no explicit power analysis was used, you should describe how you decided what sample (replicate) size (number) to use

Please outline where this information can be found within the submission (e.g., sections or figure legends), or explain why this information doesn't apply to your submission:

For the data that was analyzed using mixed-effects models, no explicit power analysis was performed beforehand; sample size was determined in advance to be no fewer than 5-6 animals per group as is standard in the field.

For the *in vivo* data, power analysis from pilot experiments suggested $n = 5$ mice were required to detect a difference in temperature threshold for seizure induction of 1 degree C (for $\alpha = 0.05$; power = 0.8). This preliminary data was not shown.

The "*n*" for all experiments (cells; imaging fields; slices; mice) is included in the Results and/or Figure legend.

Replicates

- You should report how often each experiment was performed
- You should include a definition of biological versus technical replication
- The data obtained should be provided and sufficient information should be provided to indicate the number of independent biological and/or technical replicates
- If you encountered any outliers, you should describe how these were handled
- Criteria for exclusion/inclusion of data should be clearly stated
- High-throughput sequence data should be uploaded before submission, with a private link for reviewers provided (these are available from both GEO and ArrayExpress)

Please outline where this information can be found within the submission (e.g., sections or figure legends), or explain why this information doesn't apply to your submission:

eLife Sciences Publications, Ltd is a limited liability non-profit non-stock corporation incorporated in the State of Delaware, USA, with company number 5030732, and is registered in the UK with company number FC030576 and branch number BR015634 at the address 1st Floor, 24 Hills Road, Cambridge CB2 1JP | August 2014

The "*n*" for all experiments refers to independent biological replication. We explicitly report detailed information regarding the number of cells, imaging fields, slices, and animals, used for all imaging experiments, to make clear what replicates may have shared sources of variability.

There was no exclusion of outliers.

Criteria for data exclusion/ inclusion is stated clearly in the Results and in the Methods sections.

Statistical reporting

- Statistical analysis methods should be described and justified
- Raw data should be presented in figures whenever informative to do so (typically when N per group is less than 10)
- For each experiment, you should identify the statistical tests used, exact values of N, definitions of center, methods of multiple test correction, and dispersion and precision measures (e.g., mean, median, SD, SEM, confidence intervals; and, for the major substantive results, a measure of effect size (e.g., Pearson's r, Cohen's d)
- Report exact p-values wherever possible alongside the summary statistics and 95% confidence intervals. These should be reported for all key questions and not only when the p-value is less than 0.05.

Please outline where this information can be found within the submission (e.g., sections or figure legends), or explain why this information doesn't apply to your submission:

All statistical analysis methods are described and justified. Raw data is presented in figures whenever feasible. P-values are reported exactly. This information can be found in the results, figure legends, and methods sections.

(For large datasets, or papers with a very large number of statistical tests, you may upload a single table file with tests, Ns, etc., with reference to sections in the manuscript.)

Group allocation

- Indicate how samples were allocated into experimental groups (in the case of clinical studies, please specify allocation to treatment method); if randomization was used, please also state if restricted randomization was applied
- Indicate if masking was used during group allocation, data collection and/or data analysis

Please outline where this information can be found within the submission (e.g., sections or figure legends), or explain why this information doesn't apply to your submission:

Samples were allocated into groups by genotype and mouse age. Masking was used during data collection (as feasible), and for all data analysis.

Additional data files ("source data")

- We encourage you to upload relevant additional data files, such as numerical data that are represented as a graph in a figure, or as a summary table
- Where provided, these should be in the most useful format, and they can be uploaded as "Source data" files linked to a main figure or table

- Include model definition files including the full list of parameters used
- Include code used for data analysis (e.g., R, MatLab)
- Avoid stating that data files are "available upon request"

Please indicate the figures or tables for which source data files have been provided:

All data generated or analyzed during this study are included in the manuscript and supporting files and has been uploaded to the data repository G-Node here: https://gin.g-node.org/GoldbergNeuroLab/Mattis-et-al-2022

Source data for all Figures (1-8) and Tables (1-2) is listed below has been included in the supplementary files, uploaded to G-Node, and has been made available without restriction. This includes:
- Figure 1: quantification of spikes, associated with panels G, H, I
- Figure 2 and associated Figure 2 – Figure supplements: quantification of imaging data (reported by cell, reported by field, and summarizing field responses to PTX)
- Figure 3 and associated Figure 3 – Figure supplements
- Figure 4 and associated Figure 4 – Figure supplements: quantification of effect of Hm1a wash-in on imaging data (reported by cell and by field)
- Figure 5 and associated Figure 5 – Figure supplements: quantification of patch recording data (young adult and early postnatal timepoints)
- Figure 6: quantification of seizure data
- Figure 7: quantification of seizure data
- Figure 8: quantification of PV and SST optogenetic activation plus imaging data (reported by cell and by field)
- Table 1: data summary and raw data files for all quantification of DG GC intrinsic properties
- Table 2: data summary and raw data files for all quantification of DG GC intrinsic properties

Code used for analysis is also provided via a GitHub repository: https://github.com/GoldbergNeuroLab/Mattis-et-al.-2022
- Matlab code used for analysis of 2P imaging data
- Matlab and Python code used for analysis of single cell electrophysiologic data
- Python code used for quantification of EPSCs and IPSCs
